# A192 CHANGING TRENDS IN ARTHRITIS PREVALENCE AMONG INDIVIDUALS WITH INFLAMMATORY BOWEL DISEASE: INSIGHTS FROM A POPULATION-BASED STUDY

**DOI:** 10.1093/jcag/gwae059.192

**Published:** 2025-02-10

**Authors:** E Crowley, E Kuenzig, J Widdifiled, E I Benchimol, M Lam, V Jairath, S Rohekar, L Targownik, M Watson, R Berard

**Affiliations:** Pediatrics - Gastroenterology, Hepatology & Nutrition, Western University, London, ON, Canada; The Hospital for Sick Children, Toronto, ON, Canada; University of Toronto, Toronto, ON, Canada; University of Toronto, Toronto, ON, Canada; London Health Sciences Centre, London, ON, Canada; Pediatrics - Gastroenterology, Hepatology & Nutrition, Western University, London, ON, Canada; Pediatrics - Gastroenterology, Hepatology & Nutrition, Western University, London, ON, Canada; University of Toronto, Toronto, ON, Canada; Pediatrics - Gastroenterology, Hepatology & Nutrition, Western University, London, ON, Canada; Pediatrics - Gastroenterology, Hepatology & Nutrition, Western University, London, ON, Canada

## Abstract

**Aims:**

The burden of arthritis among individuals with inflammatory bowel disease (IBD) is poorly understood. We describe changes over time in the annual prevalence of inflammatory arthritis (IA) and musculoskeletal (MSK)-related physician encounters among individuals with IBD.

**Methods:**

We conducted a retrospective repeated cross-sectional study using Ontario population-based health administrative data. We used the Ontario Crohn’s and Colitis Cohort which comprises all IBD patients derived from health administrative data using validated age-specific case-identification algorithms. Individuals diagnosed with IBD between April 01, 2003, and March 31, 2020 (population denominator) were followed from their first IBD code (index date) until they died or were lost to follow-up (out-migrated/lost health care coverage), or until the end of available follow-up data (March 31, 2020). We identified the cumulative prevalence of inflammatory arthritis (≥1 hospitalization/ED encounter or ≥2 physician billing claims with IA-related diagnosis codes with ≥1 by a rheumatologist within 365 days). Separately we identified the annual number of individuals with ≥1 hospitalization/ED encounter/physician billing claim with any non-trauma related MSK-specific diagnosis codes. The annual age- and sex-standardized cumulative prevalence of both IA and MSK among individuals living with IBD each year were determined.

**Results:**

Over the study period, the number of individuals living with IBD increased from 54,283 in 2003 to 108,857 in 2020; the number of children <18yrs increased from 1,547 to 2,667. Among all ages, the age/sex standardized cumulative IA prevalence within the IBD cohort increased from 6.8% (95% CI 6.5-7.2%) in 2003 to 15.2% (95%CI 15.0-15.8%) by 2020 (Figure 1). IA was slightly more common among those with Crohn’s than ulcerative colitis (17.4% vs 13.2%, respectively). By 2020, IA prevalence was 6.8% among children/youth <18yrs, 17.4% among those 18-64yrs, and 23.1% among those ≥65yrs. Overall, crude annual prevalence of an MSK-related encounter remained relatively stable from 30.6% in 2003 to 27.7% in 2020.

**Conclusions:**

This study is the first to provide population-level estimates of IA and MSK-related conditions in people with IBD. The cumulative prevalence of IA among individuals with IBD has steadily increased over time, particularly among those ≥65years, while MSK-related encounters have not. This may be related to increased physician recognition of IA or increased access to care and diagnosis. These findings have implications for healthcare costs and utilization, given the specialized care and expertise required to manage IA in the context of complex, comorbid conditions like IBD.

**Funding Agencies:**

CCC

